# Assessment of total sialic acid and lipid-bound sialic acid in management of brain tumors

**DOI:** 10.4103/0972-2327.56315

**Published:** 2009

**Authors:** Manjula Shantaram, Anjali Rao, Annaya Rao Aroor, Annaswamy Raja, Suryanrayana Rao, Flama Monteiro

**Affiliations:** Department of Biochemistry, Yenepoya Medical College, Mangalore, Karnataka, India; 1 Department of Biochemistry, Kasturba Medical College, Manipal, Karnataka, India; 2 Department of Medical Pharmacology and Physiology, University of Missouri School of Medicine, Columbia MO 65212, USA; 3 Department of Neurological Sciences, Kasturba Medical College, Manipal, Karnataka, India; 4 Department of Biochemistry, Mandya Institute of Medical Sciences, Mandya, Karnataka, India

**Keywords:** Brain tumors, lipid-bound sialic acid, total sialic acid, tumor markers

## Abstract

**Background:**

Glycoconjugate molecules expressed at the plasma membrane of mammalian cells have been reported to be associated with tumor progression. The measurement of total sialic acid (TSA) and lipid-bound sialic acid (LBSA) in the cerebrospinal fluid (CSF) is suggested to be useful for the diagnosis of brain tumors. But there are very few reports available on the serum glycoconjugate levels in patients with brain tumors.

**Objective:**

The objective of this study is to check the feasibility of using serum glycoconjugates such as TSA and LBSA as tumor markers in brain tumor patients.

**Materials and Methods:**

Colorimetric estimation of TSA using diphenylamine was done on 100 patients with intracranial tumors; follow-up study was carried out in 24 cases. The LBSA fraction was isolated from the serum of 68 brain tumor patients and evaluated using phosphotungstic acid and resorcinol; follow-up study was done on 23 patients. The various types of brain tumors included in this study were glioma, meningioma, and acoustic neurinoma as well as some other types such as medulloblastoma, secondary tumors, and craniopharyngioma.

**Results:**

There was no significant difference between the TSA and LBSA concentrations seen in pretreatment or post-treatment cases and that seen in control subjects.

**Discussion:**

TSA and LBSA do not have the ability to discriminate between benign and malignant brain tumors. TSA and LBSA appear to be tumor markers of very limited value in patients with brain tumors.

## Introduction

Cell surface glycoconjugates are considered to be important in relation to cancer because many of the altered properties of cancer cells are expressed at the cell surface. Warren and Buck[1] had observed that only traces of sialofucosyl glycopeptides, which is characteristic of tumor tissue, are found in the serum of healthy subjects, whereas it is found in high concentrations in malignant transformed cells.

Glycoconjugate molecules expressed at the plasma membrane of mammalian cells have been also reported to be associated with tumor progression. Growth factor receptors and glycoconjugate molecules are able to interact with each other and this interaction usually results in modulation of growth factor receptor–mediated signaling and of the biological function of the cell. The use of glycoconjugates or their derivatives may represent a new approach to the modulation of the proliferative behavior of tumors – such as brain tumors – that overexpress growth factor receptors.

Measurements of protein-bound carbohydrates have been used as an index of glycoprotein levels. Sialic acid (SA), a family of acetylated derivatives of neuraminic acid, is widely distributed in mammals, usually occurring as a terminal component at the nonreducing end of a carbohydrate chain of glycoproteins and glycolipids. As sialic acid occupies the terminal position, any change in the glycoprotein will effect a change in sialic acid and *vice versa*.

Increased density of sialic acid at the cell surface of malignant or transformed cells has been reported from studies of human systems and various malignant tumors. There are many reports on the elevation of total SA (TSA) in malignancy. In head and neck cancer, serum SA is a useful parameter.[2] Serum SA levels in oral and maxillofacial malignancy[3] can be useful in monitoring therapy. Increased SA concentrations occur in gynecological cancer;[4] cancers of lung,[5] colon,[6] and ovaries;[4] urologic cancer;[7]x melanoma;[8] etc. Monti *et al*.[9] have reported that the measurement of SA was not of clinical benefit in breast carcinoma.

Lowered levels of sialic acids were found in brain tumor tissues when compared to the levels seen in normal brain.[10] Other workers have suggested that the average concentration of serum SA increased with increase in the malignancy in brain tumors.[11] Marth *et al*.[12] reported that there was a significant difference between the average serum SA concentrations of benign and malignant brain tumors.

Interest in sialo-glycolipids as markers in cancer was generated by the discovery that circulating levels of these components were elevated in tumor-bearing animals in a pattern consistent with the concept that the lipid-bound sialic acid (LBSA) was of tumor origin.[13] Increase in serum/plasma LBSA has been reported in cancer patients in general[14] and in patients with gynecological cancer,[15] urologic cancer,[7] melanoma,[8] breast cancer,[16] bladder carcinoma,[17] and thyroid cancer.[18] Alterations in sialo-glycolipids have also been reported in brain tumors.[19]

Kakari *et al*.[20] suggested that the measurement of TSA and LBSA in the CSF should prove useful for the diagnosis of brain tumors. But there are very few reports available on the serum TSA and LBSA levels in brain tumors. Therefore, in the present study, TSA and LBSA estimations were carried out in the serum of brain tumor patients.

## Materials and Methods

### Study subjects

Patients with brain tumors, between 10 to 75 years of age, were the subjects in this prospective study carried out between December 1988 and December 1993 at Kasturba Medical College, Manipal. All were histopathologically confirmed for their neurological status. There were 60 male and 40 female patients for TSA estimation; follow-up was done for 24 cases. In case of LBSA, 68 patients with intracranial neoplasms were selected before surgical therapy. There were 45 male and 23 female patients; 23 cases were followed up. TSA and LBSA levels were estimated as per the availability of serum samples.

The various types of brain tumors included in this study were primary tumors such as glioma, meningioma, and acoustic neurinoma, as well as other types such as medulloblastoma, secondary tumors, and craniopharyngioma.

### Sample collection

Blood was collected by venipuncture prior to surgery from the patients as well as from age- and sex-matched healthy volunteers (TSA, *n* = 37; LBSA, *n* =28). The serum was separated, centrifuged, and stored at −70°C. Further serum samples were obtained from these patients when they reported for follow-up between 6–20 weeks.

TSA was estimated using cysteine hydrochloride.[21] In this method of Winzler, a protein precipitate of serum containing SA reacts with diphenylamine, producing a purple color, which is quantitatively measured in a spectrophotometer at 530 nm.

The LBSA fraction was isolated and estimated in the serum[22] by the method of Katopodis and Stock; in this method, LBSA is extracted with chloroform-methanol and evaluated using phosphotungstic acid and resorcinol reagent. The blue color that developed was measured at 580 nm.

Statistical analysis was carried out using Student's ‘*t*’ tests and one way analysis of variance (ANOVA). A *P*-value of <0.001 was considered extremely significant. Variation among column means, it is significantly greater than expected, by chance. Bonferroni multiple comparisons test: If the value of ‘*t*’ is greater than 2.792, then the *P* < 0.05.

## Results

The mean values of serum TSA and LBSA found in patients with various tumors of the brain and in healthy controls are shown in Table 1 and Table 3. There was no significant difference between the mean values [Tables 2 and 4] in patients and in healthy controls (*P* > 0.05).

**Table 1:** Levels of TSA in pretreatment cases of brain tumors (mean ± SD)

Clinical condition	TSA (mg/dl)
Control (n = 37)	55.0 ± 15.4
Glioma (n = 46)	52.0 ± 16.9
Meningioma (n = 23)	57.7 ± 23.1
Acoustic neurinoma (n = 14)	54.9 ± 15.0
Other types (n = 17)	55.5 ± 12.3

Number of patients in parentheses. n = Number of samples

**Table 2:** Comparison of TSA levels in various groups

Comparison of groups	*P* value
Control vs glioma	>0.05
Control vs meningioma	>0.05
Control vs acoustic neurinoma	>0.05
Control vs other types	>0.05

**Table 3:** Levels of LBSA in pretreatment cases of brain tumors (mean ± SD)

Clinical condition	LBSA (mg/dl)
Control (n = 28)	23.1 ± 16.3
Glioma (n = 30)	26.5 ± 15.2
Meningioma (n = 15)	27.4 ± 15.2
Acoustic neurinoma (n = 7)	25.4 ± 17.9
Other types (n = 16)	29.6 ± 15.5

Number of patients in parentheses. n = Number of samples

**Table 4:** Comparison of LBSA levels in various groups

Comparison of groups	*P* value
Control *vs* glioma	>0.05
Control *vs* meningioma	>0.05
Control *vs* acoustic neurinoma	>0.05
Control *vs* other types	>0.05

**Figure 1:**
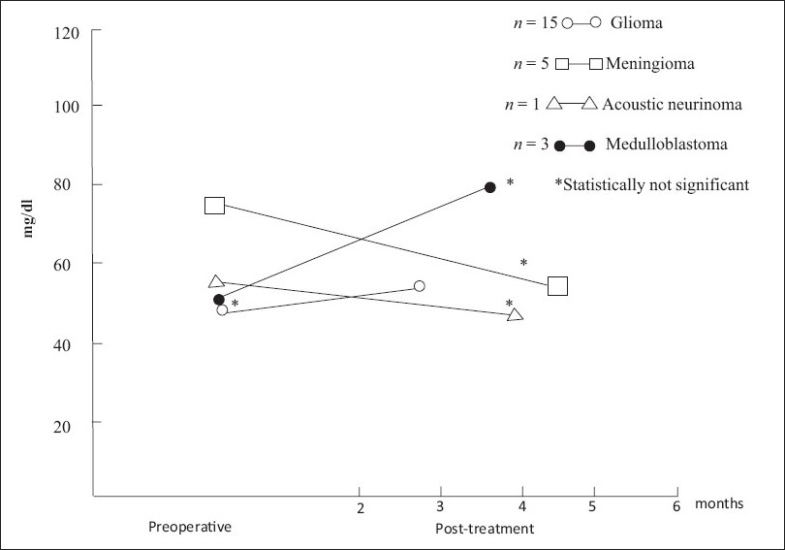
Total sialic acid in preoperative and post-treatment cases (mean)

**Figure 2:**
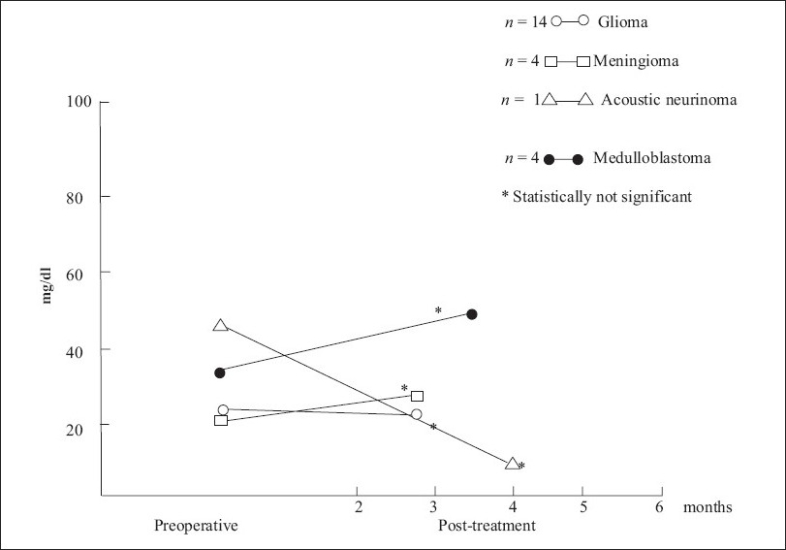
Lipid-bound sialic acid in preoperative and post-treatment cases (mean)

TSA and LBSA levels in post-treatment cases remained in the normal range when compared to that of controls as well as to their preoperative values [Figures 1 and 2].

A comparison of the mean TSA and LBSA concentrations in patients with benign and malignant tumors with that seen in the control group showed no significant difference between the groups (*P* > 0.05) [Table 5].

**Table 5:** Comparison of serum TSA and LBSA in benign and malignant tumor cases (mean ± SD)

Sialic acid	Control	Benign cases	Malignant cases	*P* value
TSA (mg/dl)	55.0 ± 15.4 (n = 37)	56.9 ± 18.6 (n = 48)	51.9 ± 16.4 (n = 52)	>0.05 ns
LBSA (mg/dl)	23.1 ± 16.3 (n = 28)	25.9 ± 15.0 (n = 33)	28.6 ± 15.5 (n = 35)	>0.05 ns

n = Number of samples; ns = not significant (one way ANOVA).

## Discussion

Winzler[21] suggested that in view of the multiplicity and heterogeneity of serum glycoproteins, it is likely that different components may have different sites of origin and reflect different pathological processes. The SA–containing glycoproteins and gangliosides of the cell membrane are known to undergo significant alterations during malignant transformation. Determination of the TSA in the serum reflects the level of glycoconjugates. The mechanism whereby SA in the serum and in the CSF is elevated has been suggested to be selective cleavage of surface glycoproteins that may result in the ‘shedding’ of glycoproteins, which eventually find their way into the blood circulation and the CSF.[12]

Flaschka *et al*,[11] found that the average concentration of serum SA was increased with increasing malignancy and that the patients with non-tumorous diseases of CNS with brain tissue lesions had extremely high values. Further, Marth *et al*,[12] found a significant difference between the average serum SA concentrations in patients with benign brain tumors and those with malignant brain tumors. However, Nakamura *et al*,[10] observed that all tumor tissues contained lower SA than normal brain tissue.

Gangliosides are a family of SA–containing glycosphingolipids that mediate cell adhesion; they modulate cell growth through their effect on growth factor receptor tyrosine kinases. In addition, it is demonstrated that some glycosphingolipids, particularly gangliosides, play an essential role in defining cell motility through their interaction with integrins and tetraspanin CD9 or CD82.[23–25]

Altered composition and concentration of gangliosides were found in human gliomas when compared to normal grey and white matter of the brain. The major gangliosides GM1, GD1a, and GT1b were markedly reduced in tumor tissue and, in contrast, there was an increase of gangliosides GM3 and GD3, which often appeared as the dominant ones. Moreover, the mono- and oligosialylated gangliosides present in brain tumor tissue were not detected in normal brain.[26] TSA and LBSA levels were found to be significantly increased in the CSF of patients with glioma.[20]

In the present study, TSA and LBSA levels were found to be in the normal range in the preoperative and post-treatment serum samples of brain tumor patients. LBSA elevations are relatively nonspecific with respect to the type of cancer and thus would seem to be of limited value for routine cancer detection.[27] Stratton *et al*,[28] reported that LBSA was a nonspecific marker in gynecological malignancies. It is generally considered that human serum contains no free SA and that 90% of the serum SA is bound to the α- and β-globulins. LBSA levels are observed to be in the normal range, probably due to the greater binding of serum SA to proteins than to lipids. The LBSA level may probably not be a useful marker in brain tumors; it was found to be of very limited value in this study and other authors have reported similar findings in other malignancies.[28,29]

However, tumor cells with a pharmacologically decreased concentration of gangliosides produce fewer tumors in mice than do untreated cells, suggesting that pharmacologic depletion of gangliosides should be explored further as a therapeutic approach to cancer.[30]
